# Research Hotspots and Trends of Virtual Reality Intervention for Stroke: Bibliometric Analysis

**DOI:** 10.2196/65993

**Published:** 2025-04-16

**Authors:** Yixin Wei, Yuan Chen, Runting Ma, Yitong Qiu, Wei Su, Li Zhang, Qiang Gao

**Affiliations:** 1 Rehabilitation Medicine Center and Institute of Rehabilitation Medicine, West China Hospital, Sichuan University, 37 Guoxue Lane, Chengdu, 610041, China, 86 18980605992; 2 Key Laboratory of Rehabilitation Medicine in Sichuan Province, West China Hospital, Sichuan University, Chengdu, China; 3 Department of Pharmacy, Evidence-Based Pharmacy Center, West China Second Hospital, Sichuan University, Chengdu, China

**Keywords:** virtual reality, stroke, bibliometric analysis, hotspots, research trends

## Abstract

**Background:**

Virtual reality (VR) is a rapidly developing technology that has gained significant traction in the treatment and rehabilitation of individuals with stroke. Research on VR-based stroke treatment has garnered increasing attention.

**Objective:**

The aim of this study is to present a bibliometric analysis of VR for stroke studies to identify the application status, research hotspots, and emerging trends and guide future scientific research.

**Methods:**

We included studies and reviews on the topic of VR-based stroke treatment and rehabilitation from 1999 to 2023 were retrieved from Web of Science Core Collection database. Citespace 6.3.1 and VOSviewer 1.6.20 software was used for the visual analysis of publications, institutions, authors, journals, citations, and Scimago Graphica software was used for the geographic visualization of published countries or regions.

**Results:**

Our study analyzed 1171 papers on VR-based stroke rehabilitation published between 1999 and 2023, revealing a gradual increase in annual publications over the past 2 decades, peaking at 154 in 2022. North America and Western Europe were identified as major contributors, with significant input from their institutions, researchers, and publications. The Journal of NeuroEngineering and Rehabilitation emerged as the leading journal in this field, while Calabrò Rocco Salvatore was recognized as the most prolific author, focusing on the neurophysiological impacts of VR on patients with stroke. Keywords with notable citation bursts, such as “environment,” “trial,” “arm,” and “motor learning,” highlighted the core research themes in this domain.

**Conclusions:**

Our study provides valuable insights into the current research hotspots and emerging trends in VR-based stroke treatment and rehabilitation. Current research primarily focuses on evaluating the effectiveness of VR in improving upper limb function and balance in patients with stroke. Future directions are shifting towards integrating VR with rehabilitation techniques, such as physiotherapy and occupational therapy, while advancements in VR technology continue to garner increasing attention.

## Introduction

Stroke is a significant contributor to mortality and morbidity globally, ranking as the leading cause of permanent acquired disability [1]. In 2016, there were 80.1 million cases of stroke worldwide, and this figure is likely to increase further in the future due to the growth of the aging population [2]. A significant proportion of patients who have suffered from stroke report a range of impairments to motor, sensory, cognitive, and visual function [3]. This results in a considerable number of stroke survivors being unable to perform activities of daily living, participate fully in society, or interact socially in an effective manner [3]. The accumulating evidence from research indicates that rehabilitation is the most effective means of reducing disability and a vital component of stroke management [4,5]. The conventional neurorehabilitation approach, which involves high-intensity, repetitive, and task-specific practice, has demonstrated a curative effect on enhancing the functional abilities of individuals with stroke [6,7]. However, these rehabilitation techniques are costly and inconvenient due to their reliance on professional expertise and substantial resource requirements, particularly in the context of the global pandemic of COVID-19 [8,9]. It follows that there is a pressing requirement for a novel and efficacious approach to stroke management.

Virtual reality (VR) can be defined as an interactive, computer-generated environment that simulates the real world [10]. This enables interaction with VR images and sounds, which prompt user responses and deliver immediate performance feedback. This interaction is facilitated through a variety of devices, including computers, mobile screens, and head-mounted displays, which collectively enhance user engagement and experience. With the continuous advancement of health technologies, VR is anticipated to play a more prominent role in clinical rehabilitation [11,12]. The application of VR technology in the rehabilitation of a diverse range of diseases that emerged in the 1990s has become increasingly prevalent [10,13-15]. Over the past 60 years, the technology has undergone significant transformations. In recent years, VR has emerged as a vital tool for both assessment and intervention in clinical rehabilitation, driven by extensive research and a reduction in the costs of VR technologies [10,16,17].

VR represents a novel technology with the potential to be used with greater frequency in clinical stroke rehabilitation [6,16]. Common examples of VR on stroke neurorehabilitation are as follows: (1) manipulating VR objects exercises for upper limb motor function [18,19], (2) VR-based training system for regaining gait, balance and daily function [20], and (3) cognitive-motor exergame training based on VR program for cognitive rehabilitation [17,20]. The therapeutic benefits of VR in stroke rehabilitation are attributed to mechanisms such as task-specific repetition, strategic feedback, and embodied simulation. In addition, the enriching environmental context provided by VR therapy has been substantiated in numerous studies, highlighting its efficacy in promoting neuroplasticity and functional recovery.

In light of the growing demand for stroke rehabilitation, the increasing application of VR therapy for stroke has positioned it as a significant area of research [16,18,20,21]. Guo et al conducted a 25-year visualization analysis of VR for neurorehabilitation, which indicated that VR for stroke is a promising avenue for future research [22]. However, existing studies often suffer from complexity, lack of structure, and an incomplete understanding of the underlying therapeutic mechanisms. These limitations, coupled with the absence of an up-to-date and comprehensive bibliometric analysis, underscore the need for a systematic investigation into the scientific landscape of VR for stroke rehabilitation. Bibliometric analysis, as a cross-disciplinary field of quantitative analysis, applies mathematical and statistical methods to examine knowledge carriers. It is a widely used methodology for identifying the developmental trajectory of specific research areas [23]. By analyzing citation networks, co-citation patterns, and the distribution of academic output across regions and institutions, bibliometric analysis allows researchers to quickly grasp current research hotspots, identify influential studies, and discern emerging trends [24]. In this context, bibliometric analysis provides a valuable tool for synthesizing large volumes of literature and offering a structured, data-driven perspective on the research landscape. This bibliometric study aims to provide a comprehensive overview of the global scientific output on the use of VR for stroke rehabilitation, spanning from its inception to 2023. We used Citespace and VOSviewer software to map the intellectual structure of the field and visualize citation patterns, co-citations, and the distribution of research across countries, authors, and journals.

## Methods

### Data Collection

Web of Science (WOS) database, a renowned scientific data services platform developed by Clarivate was used to retrieve the Clarivate Journal Impact Factor (IF) for the last 5 years [25]. Meanwhile, publications with related themes from the inception (1900) to 2023 were searched from the Science Citation Index Expanded (SCIE) of the Web of Science Core Collection (WOSCC) database. SCIE is a subdatabase of WOSCC, which consists of global journals of basic science research, covering neuroscience and medical research related to the theme of this study, “VR for stroke.”

The data were obtained on March 30, 2024, from SCIE. To obtain documents explicitly using the concerning terms we performed a topical search with the query TS=(((“Virtual reality”) OR (VR)))) AND TS=(((stroke* OR brain vascular accident* OR cerebrovascular accident*) OR ((ischemi* OR infarct* OR thrombo* OR haematoma* OR Fhematoma* OR haemorrhag* OR hemorrhag*) AND (brain* OR cerebr* OR cerebell* OR intracran*))). We only selected articles and reviews in English, other document types, such as letters, commentaries, and meeting abstracts, were excluded. Finally, a total of literature records was included.

### Data Import and Deduplication

All included documents underwent peer review. We imported all bibliometric data into Endnote 20 (Clarivate) to remove duplicates. Then, we independently screened the titles, abstracts, and full texts of the included papers based on the exclusion criteria to identify eligible studies. The exclusion criteria were (1) VR intervention not present, (2) targeted conditions not related to stroke, and (3) paper theme not correlated with VR implementation in stroke neuromodulation. Finally, 1171 articles (924 articles and 247 reviews) meeting the criteria were included.

### Data Merging

After screening and verification in Endnote, relevant literature was manually selected from the WOS database. The plain text containing information about these documents was downloaded from the WOS database. After downloading the data, field items need to be manually merged. We identify 3 common scenarios requiring data merging and propose solutions for each. These scenarios include (1) different spellings or formats of the same country name, for example, “USA” and “United States of America” will be merged into “USA”; and (2) different abbreviations or variations of the same author’s name, resolved using ORCID information and author affiliation.

### Data Analysis and Visualization

After data deduplication and merging, the plain text will import into VOSviewer 1.6.18, Citespace 6.2.4, Scimago Graphica, and GraphPad Prism 10.4.0 for further analysis and visualization. Quantitative and qualitative analyses were performed and presented in this study, including the following:

First, the time trends of publication were analyzed based on the annual number of publications after applying the screening criteria. The number of publications per year and the cumulative number of publications were calculated and visualized using GraphPad Prism software. A second-order polynomial (quadratic) fit was applied to the cumulative publication data to model its trajectory and predict future trends in research publication output [26]. This approach enables the identification of growth patterns and provides insights into the potential future development of the VR for stroke [27].

Second, the country distribution of publications was analyzed using Scimago Graphica software. After data extraction and screening, the countries contributing to the research publications were identified and their publication counts were aggregated. The results were visualized using Scimago Graphica to generate intuitive and visually appealing geographic maps [28]. These maps illustrated the spatial distribution and relative contribution of each country to the field, enabling a clear understanding of the global research landscape.

Third, the distribution of journals, institutions, and authors contributing to the research was analyzed using VOSviewer. The software was used to process co-authorship, co-citation, and bibliographic coupling relationships among journals, institutions, and authors. VOSviewer extracted bibliometric metadata, including titles, authors, affiliations, and journal information. Applied clustering algorithms in VOSviewer to group related entities, identifying patterns and prominent contributors. Generated network visualizations representing nodes (journals, institutions, and authors) and links (relationships such as citations or collaborations). These visualizations provided insights into the academic landscape, highlighting significant journals, leading institutions, and key authors in the research field [29].

Fourth, to analyze keyword clustering and detect emerging trends, CiteSpace was used to process and visualize bibliometric data. A visual network of keywords was generated, with nodes representing keywords and edges representing co-occurrence relationships [30]. The combined analysis of keyword clustering and citation bursts provided a comprehensive understanding of the intellectual structure, research hotspots, and evolving trends in the field [30,31].

### Ethical Considerations

Our study falls under the category of bibliometric research. As this study is classified as bibliometric analysis, it did not require formal ethics approval. Bibliometric research involves the quantitative examination of publicly accessible scholarly publications (eg, journal articles, patents, and conference proceedings) and does not entail (1) direct interaction with human participants, (2) collection of personal, sensitive, or identifiable data, and (3) interventions in human behavior or health.

Under international guidelines (eg, the Helsinki Declaration) and institutional policies, studies of this nature are typically exempt from ethics review, as they pose no risks to privacy, autonomy, or welfare.

## Results

**Figure 1.**
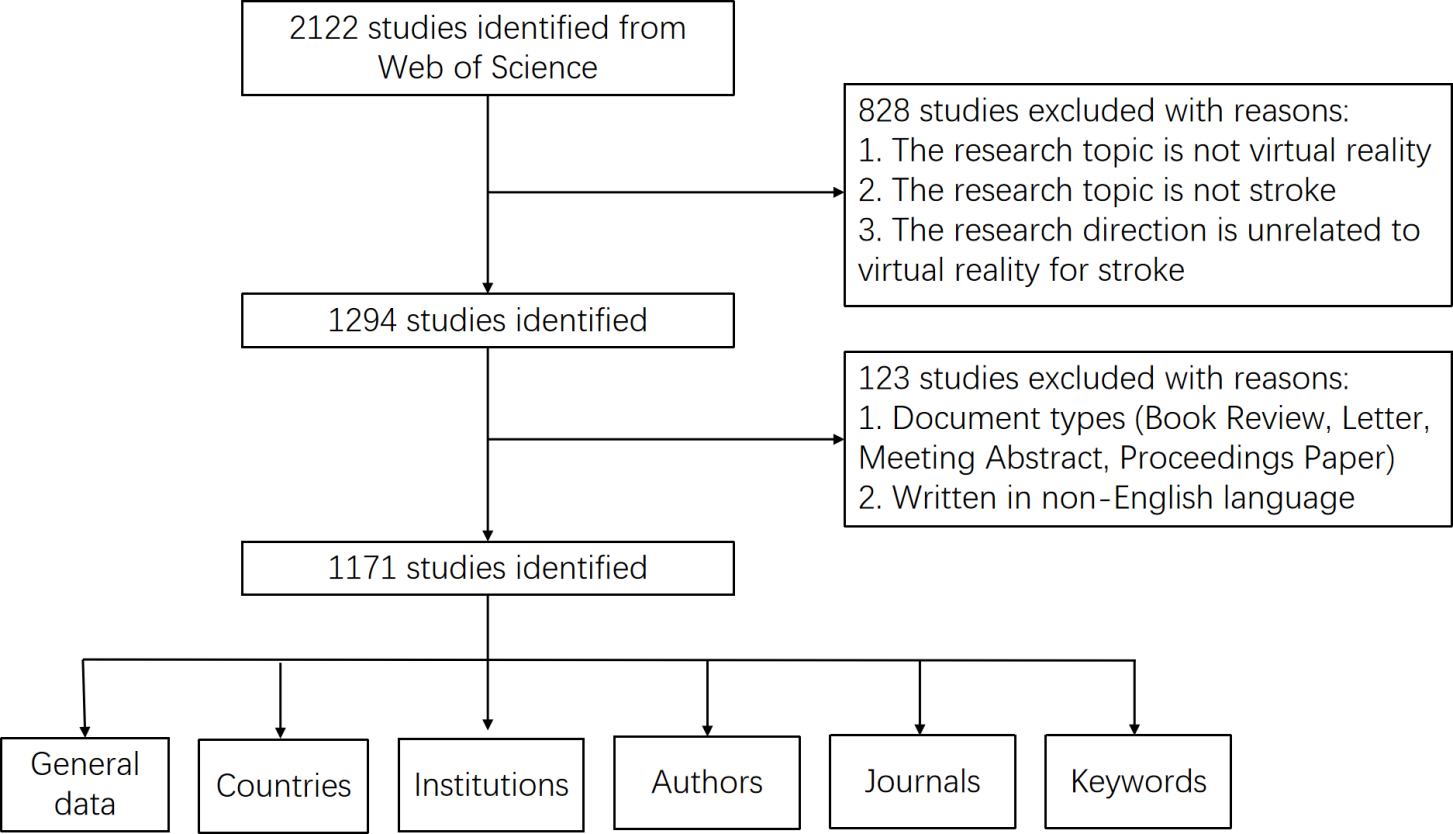
Flow chart of the bibliometric search and analysis process.

Figure 1 shows the bibliometric search and analysis flowchart.

### Time Trends of Publication

**Figure 2.**
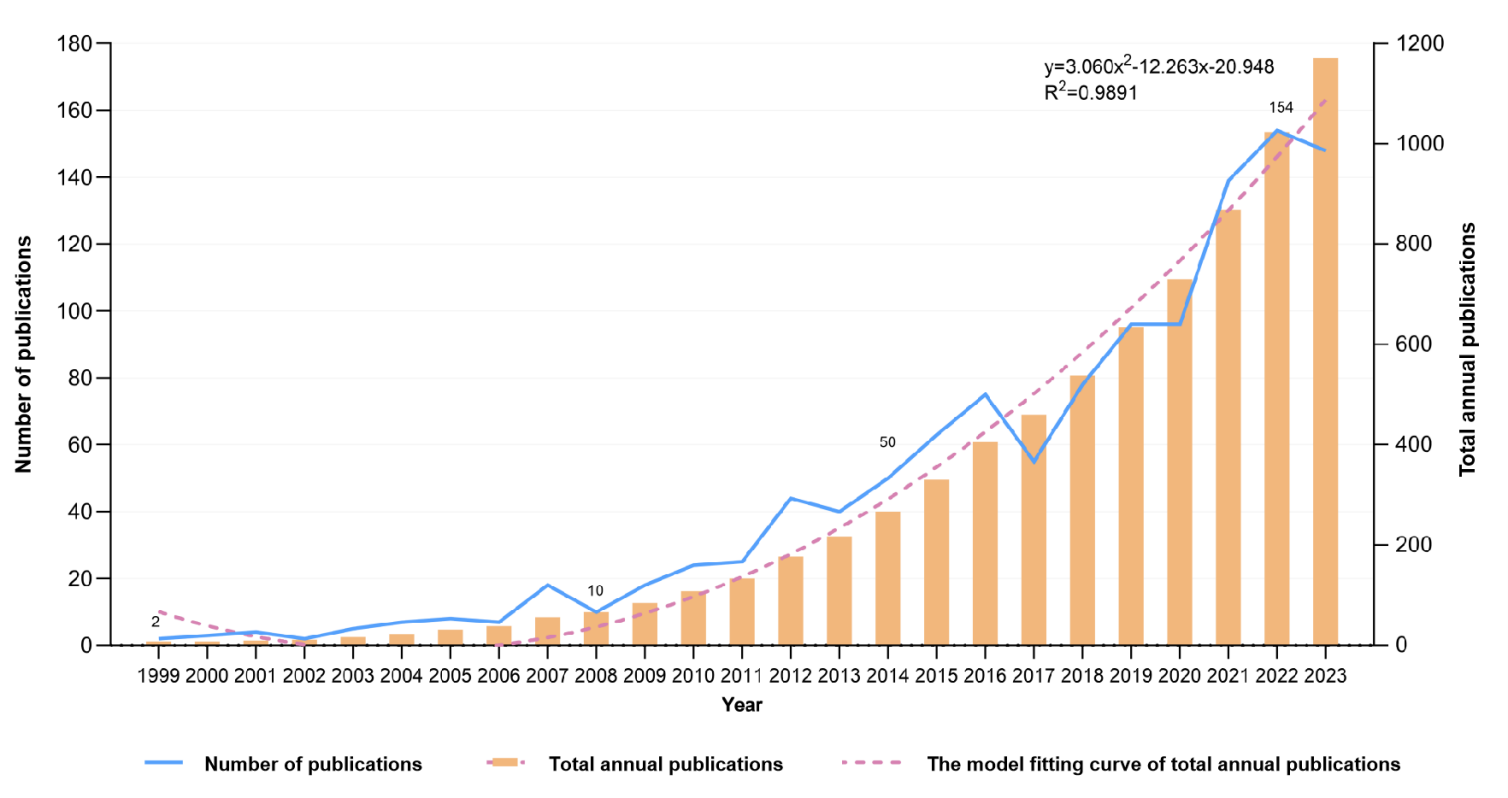
Trend of publication outputs from 1999 to 2023 on virtual reality (VR) for stroke topic.

The initial search within the WOSCC identified a total of 2122 publications. Subsequent filtration to exclude nonacademic literature types, publications not written in English, and studies outside the scope of the targeted research theme narrowed the field to 1171 eligible papers. This corpus includes 844 research articles and 327 review articles, spanning a publication period from 1999 to 2023. The temporal distribution and evolution of the annual publication volume from the inception of the database until 2022 are illustrated in Figure 2. The publication timeline has been segmented into 3 distinct phases for analysis: the infancy phase (1999‐2006), during which annual publications did not exceed 10; the slow-growth phase (2007‐2014), characterized by a steady increment in publications, rising from 10 papers in 2008 to 50 papers by 2014; and the high-growth phase (2015‐2023), where the publication rate consistently surpassed 50 papers annually, demonstrating a robust upward trajectory in research output. Furthermore, a linear regression analysis was performed to assess the growth trends in publication output, revealing a significant positive correlation with the model y=3.060x^2^−12.263x−20.948 (*R*^2^=0.9891; the closer *R*^2^ is to 1, the better the fit of the trendline), underscoring a robust expansion in scholarly activities. The analysis forecasts a sustained increase in research productivity in the area, suggesting a thriving academic interest and a promising future for further investigations.

### Distribution of Countries

**Figure 3.**
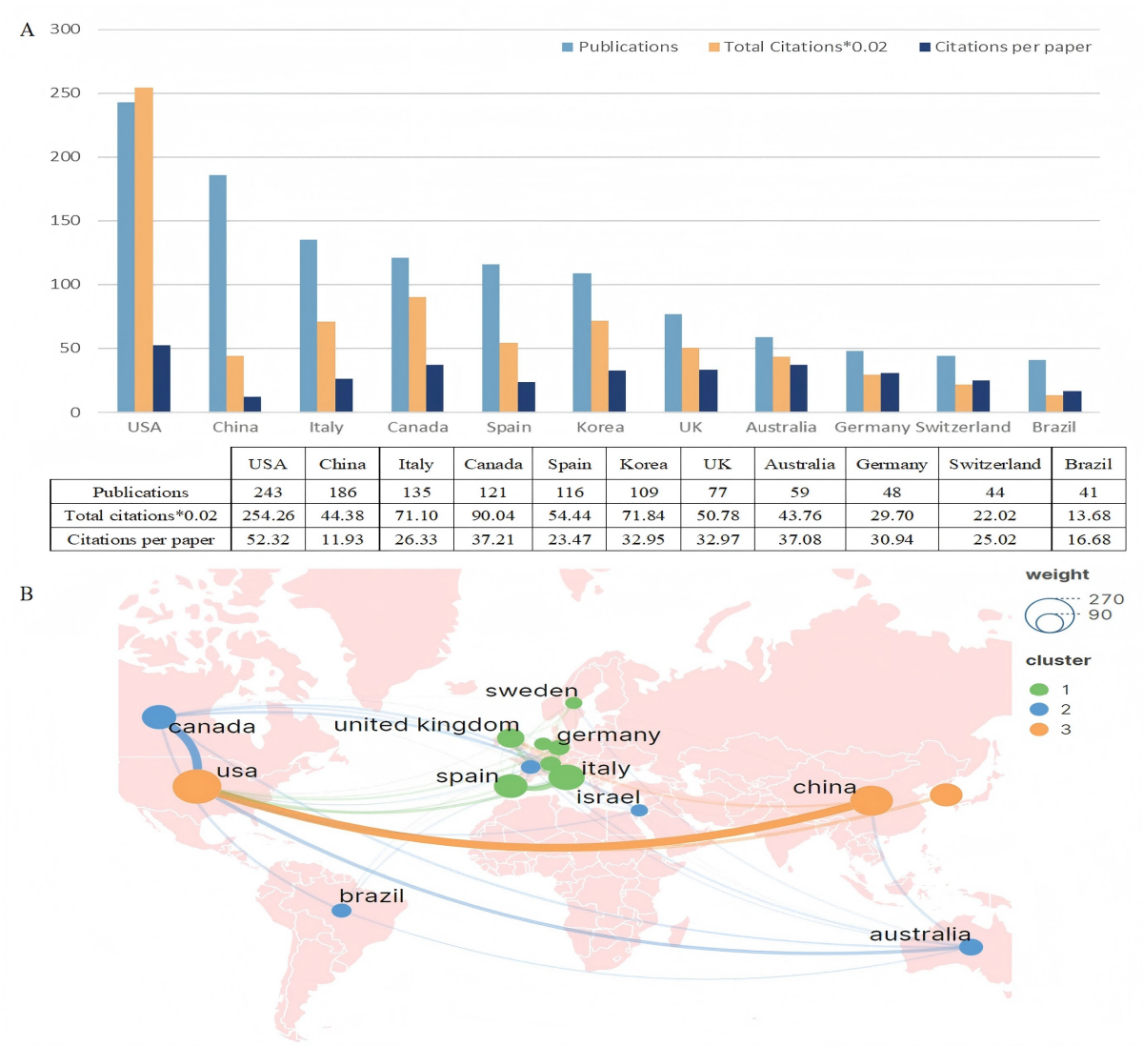
The top 11 prolific countries and international collaboration network on virtual reality (VR) for stroke research. (**A**) The number of publications, total citations, and citations per paper in the top 11 countries. (**B**) The cooperative network visualization map of countries.

Figure 3A delineates the leading 11 nations or regions based on their scholarly output in the field. The United States spearheads this list with 243 publications, closely followed by China with 186, and Italy with 135. Canada, Spain, and Korea each have contributed over 100 publications to this domain. Notably, within this group of prolific publishers, only China and Brazil are classified as developing nations, with the remainder being developed nations. Research originating from the United States not only leads in quantity but also in scholarly impact, amassing 12,713 citations, with Canada and Italy following with 4502 and 3555 citations, respectively. When examining citation efficiency, the United States remains at the forefront with an average of 52.32 citations per publication, with Canada at 37.21 and Australia close behind at 37.08. The geographical distribution of the included studies was analyzed using VOSviewer and further visualized through Scimago Graphica, facilitating the creation of a cooperative network depicted in Figure 3B. This network prominently features 3 principal clusters located in North America, Western Europe, and Eastern Asia. Among these, the strongest collaborative ties are observed between the United States and China, highlighting a significant axis of academic partnership within the field.

### Analysis of Institutions

**Figure 4.**
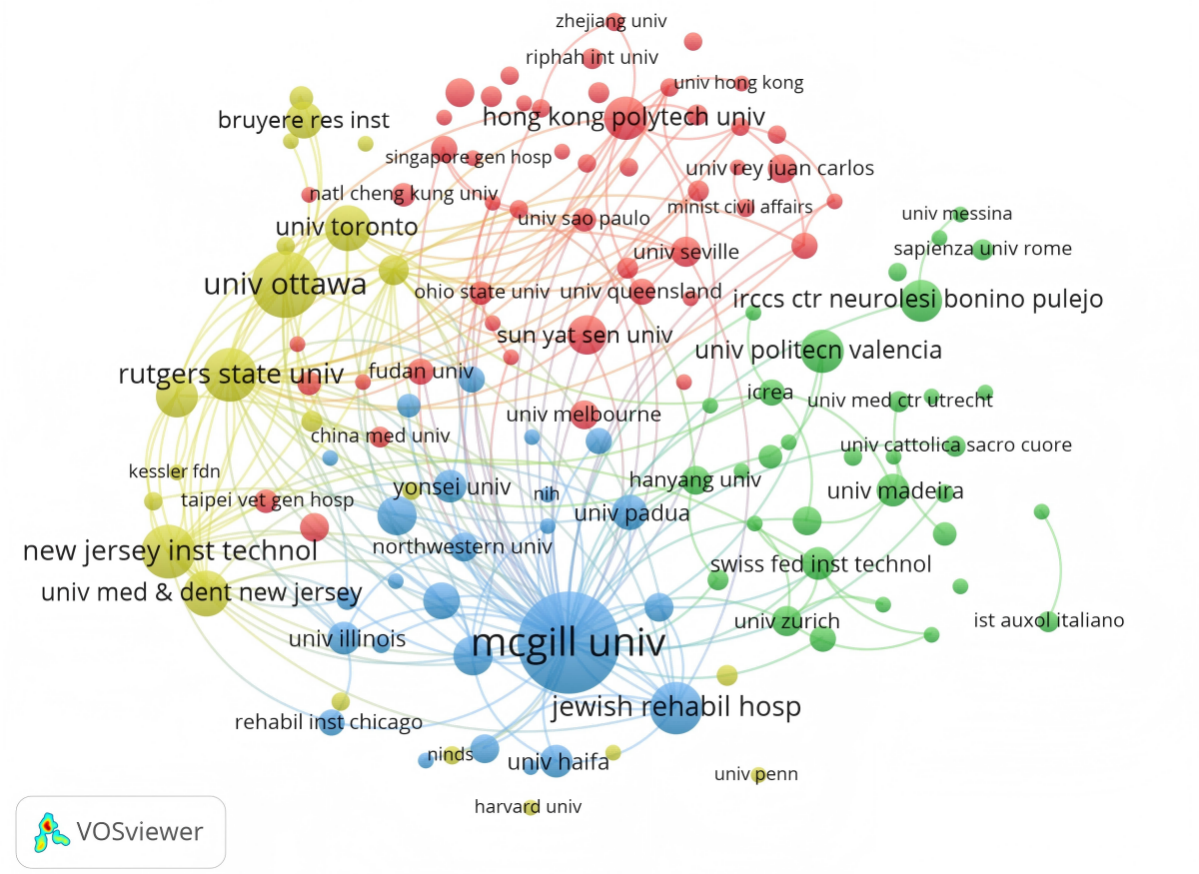
The VOSviewer density visualization map of institutions in the field of virtual reality (VR) for stroke.

The dataset reveals participation from 1916 distinct institutions in the authorship of 1171 scholarly articles. Table 1 enumerates the top 10 contributing institutions, with McGill University in Canada leading the cohort with a total of 49 publications. This is closely followed by the University of Ottawa, also in Canada, with 29 papers, and Rutgers University in the United States, contributing 22 papers. Notably, Rutgers University also boasts the highest citation count, amassing a total of 2210 citations, and an impressive average of 100.45 citations per paper. Figure 4 elucidates the interinstitutional collaborative network among the leading entities applying virtual reality to stroke research. these collaborative ties, 4 institutions represented by McGill University, University of Ottawa, Hong Kong Polytechnic University, and Istituto di Ricovero e Cura a Carattere Scientifico (IRCCS) Centro Neurolesi Bonino Pulejo have formed 4 representative networks. The connections between these networks are robust, underscoring a highly cohesive and dynamic collaborative framework. These institutions collectively exemplify a strong and well-integrated network of cooperation in advancing this field of research.

**Table 1.** The top 10 productive institutions regarding the research on virtual reality (VR) for stroke.

Rank	Institution	Country	Count	Citations
1	McGill University	Canada	49	2166
2	University of Ottawa	Canada	29	602
3	Rutgers University	United States	22	2210
4	New Jersey Institute of Technology	United States	21	1529
5	Jewish Rehabilitation Hospital	Canada	21	708
6	University of Toronto	Canada	18	1526
7	Polytechnic University of Valencia	Spain	17	436
8	Hong Kong Polytechnic University	China	17	144
9	IRCCS^a^ Centro Neurolesi Bonino Pulejo	Italy	16	453
10	Northeastern University	United States	16	389

a IRCCS: Istituto di Ricovero e Cura a Carattere Scientifico.

### Analysis of Journals

**Figure 5.**
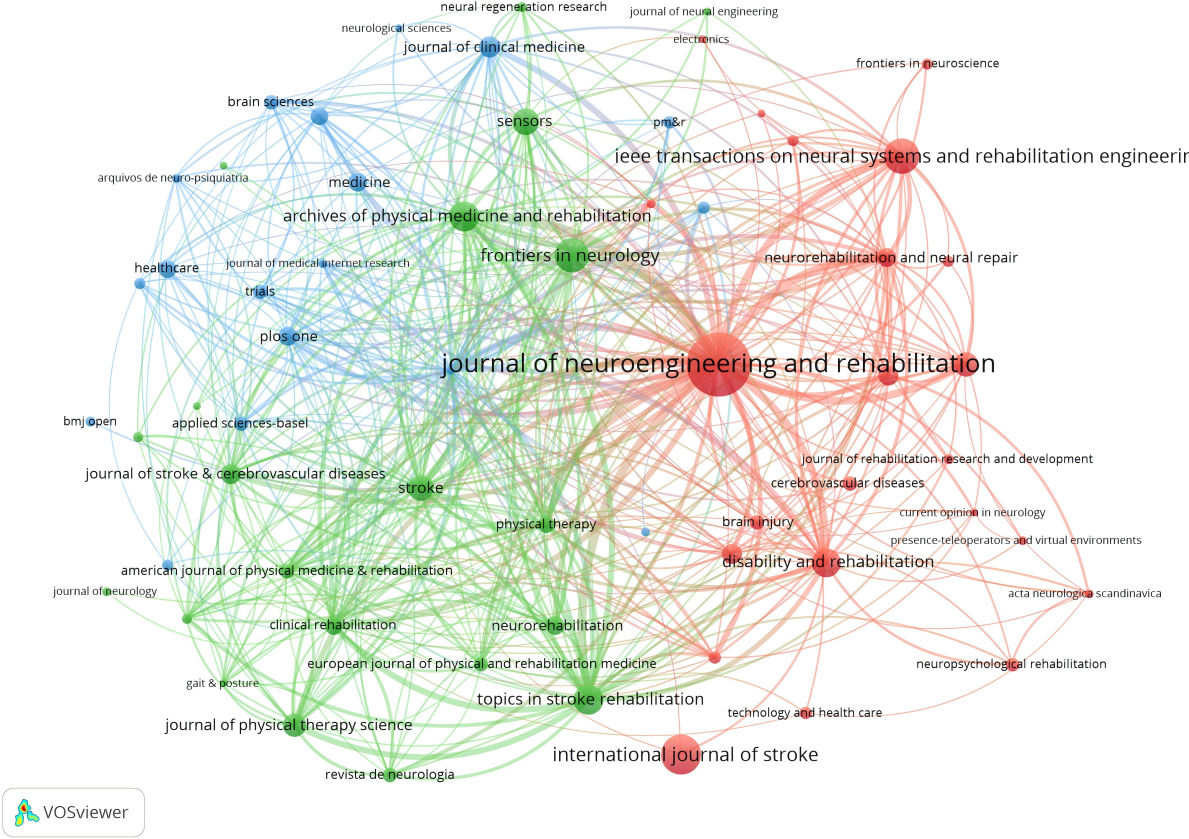
VOSviewer co-citation analysis clustering map of journal in the field of virtual reality (VR) for stroke.

This study analyzed 1171 scholarly articles published across 344 academic journals. According to Bradford’s Law, core journals are identified as those publishing more than one-third of all journal articles within a specific field. Within this area of research, 12 journals were classified as core, while 332 were deemed noncore. Table 2 indicates that the top 10 journals collectively accounted for 29.97% of the publications, contributing 351 articles. The *Journal of Neuroengineering and Rehabilitation* led with 79 publications, followed by the *International Journal of Stroke* with 42 papers, and *Institute of Electrical and Electronics Engineers (IEEE) Transactions on Neural Systems and Rehabilitation Engineering* with 37 papers. The highest IF observed over the past 5 years was 8.8, associated with the journal *Stroke*. Among the journals, 5 boasted an IF greater than 5.000, 3 had IF ranging from 3.000 to 5.000, and 2 journals recorded an IF below 3.000. The impact of scholarly publications in a research area is quantified by the number of co-citations they receive. The co-citation analysis was conducted using the VOSviewer and is presented in Figure 5. The size of the nodes represents the number of co-citations, and the lines connecting the nodes indicate co-citation relationships. With regard to the color coding used in the cluster analysis, the red cluster represents journals specializing in rehabilitation engineering, such as the *Journal of Neuroengineering and Rehabilitation* and the *IEEE Transactions on Neural Systems and Rehabilitation Engineering*. The green cluster represents academic journals in the field of neurology research, with *Stroke* and *Frontiers in Neurology* being representative examples. General medical journals, represented by *Medicine*, are classified as the blue cluster.

**Table 2.** The top 10 journals that published articles regarding the research on virtual reality (VR) for stroke.

Rank	Journal title	Country	Count	Citations	JCR^a^	IF^b^ 5 year
1	*Journal of Neuroengineering and Rehabilitation*	England	79	3145	Q2^c^	5.8
2	*International Journal of Stroke*	England	42	170	Q1	6.5
3	*IEEE*^*d*^ *Transactions on Neural Systems and Rehabilitation Engineering*	United States	37	1603	Q1	5.5
4	*Frontiers in Neurology*	Switzerland	35	418	Q2	3.9
5	*Topics in Stroke Rehabilitation*	England	29	1491	Q2	2.6
6	*Archives of Physical Medicine and Rehabilitation*	United States	29	1215	Q1	4.4
7	*Disability and Rehabilitation*	England	28	836	Q2	2.5
8	*Sensors*	Switzerland	25	209	Q2	4.1
9	*Stroke*	United States	24	1617	Q1	8.8
10	*Virtual Reality*	England	23	203	Q2	5.5

a JCR: Journal Citation Reports.

b IF: impact factor.

c Q: Quartile.

d IEEE: Institute of Electrical and Electronics Engineers.

### Analysis of Authors

**Figure 6.**
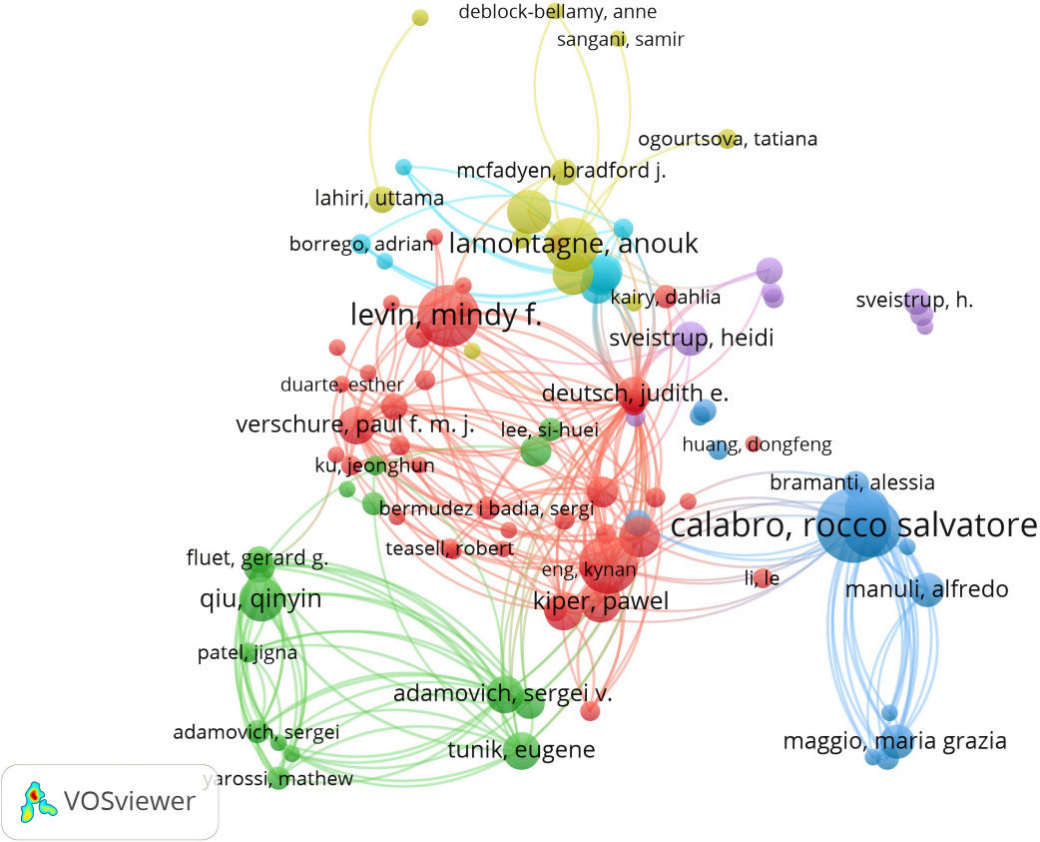
Network map of active authors contributed to virtual reality (VR) for stroke research.

A total of 5319 authors were engaged in the documents on VR in the field of stroke. Table 3 shows the top 10 most active authors and their related information. Calabrò Rocco Salvatore published 22 publications, ranking first among all authors. The majority of the top 10 authors are affiliated with Italian and Canadian research institutions. As illustrated in Table 3, Andrea Turolla from the University of Bologna was the most cited author, with 896 citations. The average number of citations per paper, Tonin Paolo from the Sant’Anna Institute was the most cited author. Furthermore, the H-index can also accurately reflect the academic achievements of authors. Levin Mindy was ranked first in H-index (50) and has the largest impact in the field. Figure 6 shows an overlay visualization of the author co-occurrence analysis generated by VOSviewer. The graph forms major clusters centered on the top 10 authors. The collaboration between them is particularly strong and occurs predominantly within the same cluster.

**Table 3.** The top 10 active authors in virtual reality (VR) for stroke research.

Rank	Author	Institution	Country	Publications	Citations	H-index
1	Calabrò, Rocco Salvatore	Scientific Institute for Research, Hospitalization and Healthcare	Italy	22	565	33
2	Levin, Mindy	McGill University	Canada	18	816	50
3	Andrea, Turolla	University of Bologna	Italy	17	896	26
4	De Luca, Rosaria	IRCCS^a^ Centro Neurolesi Bonino Pulejo	Italy	17	524	28
5	Lamontagne, Anouk	McGill University	Canada	16	304	26
6	Qiu, Qinyin	Rutgers University	United States	13	392	14
7	Naro, Antonino	IRCCS Centro Neurolesi Bonino Pulejo	Italy	13	328	29
8	Fung, Joyce	McGill University	Canada	13	246	33
9	Tonin, Paolo	Sant'Anna Institute	Italy	12	828	33
10	Pawel Kiper	IRCCS San Camillo Hospital	Italy	12	472	16

a IRCCS: Istituto di Ricovero e Cura a Carattere Scientifico.

### Analysis of Keywords

**Figure 7.**
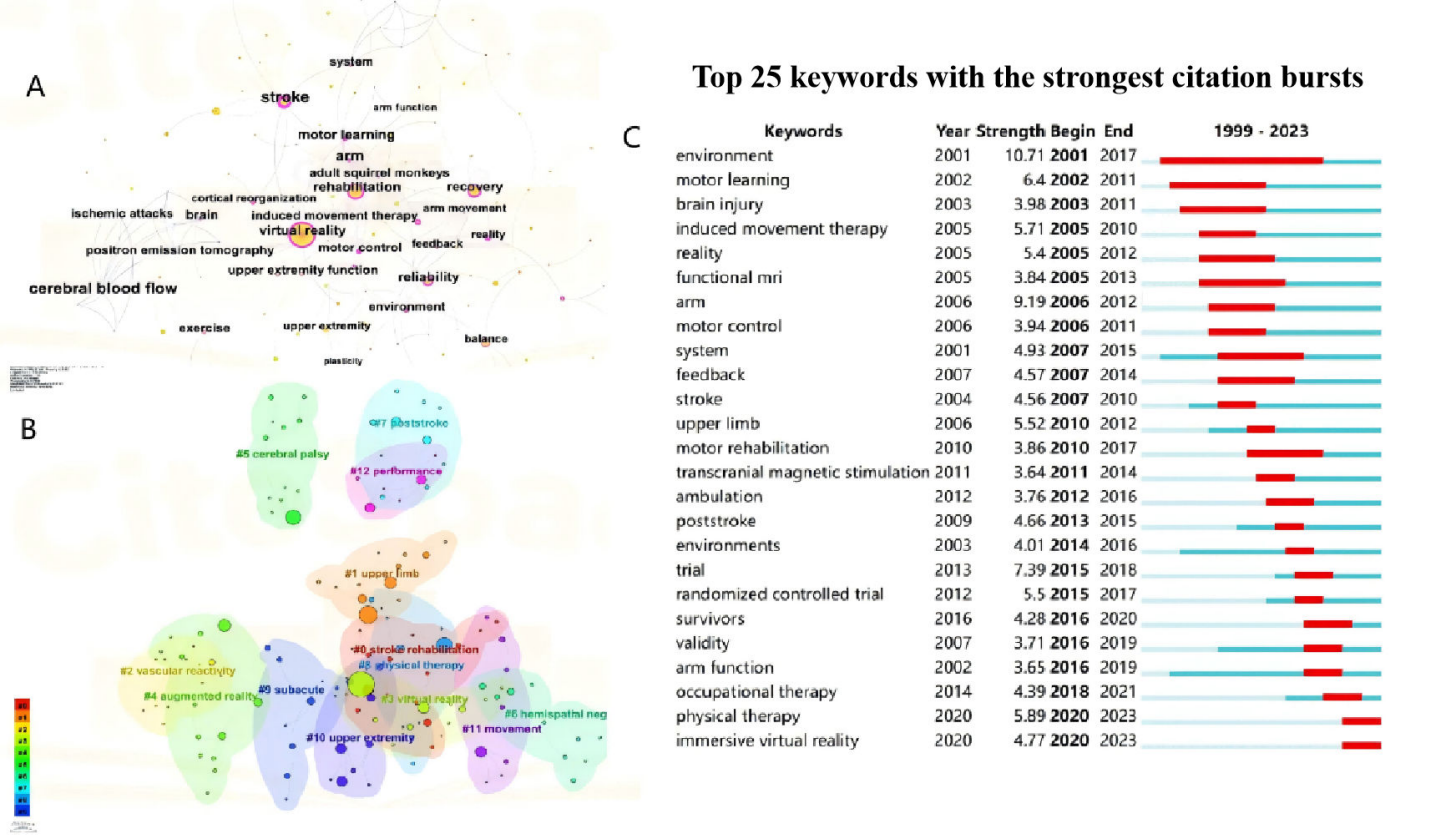
Analysis of keywords related to publications on virtual reality (VR) for stroke field from 1999 to 2023. (**A**) The keyword co-occurrence network map. (**B**) The keyword cluster map. (**C**) The top 25 keywords with the strongest citation bursts.

Keywords are words that have a significant meaning within the context of a paper. The identification of high-frequency or emergent keywords can provide insight into the current topics of interest and potential future research frontiers. As shown in Figure 7A, the top 5 most frequent keywords are virtual reality, rehabilitation, recovery, stroke, and upper limb. According to the different types of keywords, the keywords can be categorized into 13 clusters, as shown in Figure 7B. The #0, #3, #4, and #8 clusters mainly describe the main forms and means of using VR in stroke rehabilitation. The #1, #10, #11, and #12 clusters primarily delineate the principal objective of VR in stroke rehabilitation is the enhancement of upper limb function and performance. The other clusters represent the application of VR to different periods of stroke, as well as other dysfunctions of stroke.

Citation burst analysis is a valuable tool that can provide insightful analysis of specific areas of research for specific year hotspots and trends. Citespace is used to generate the top 25 keywords with the strongest bursts from 1999 to 2023, with the results presented in Figure 7C. Among these keywords, environment exhibits the highest burst intensity. Arm function, physical therapy, occupational therapy, and immersive virtual reality are the most recent emergent keywords, which indicates the recent research direction.

## Discussion

### Overview of the Results

In this bibliometric study, 1171 papers focusing on VR for stroke were included and visualized by VOSviewer and Citespace to display the research hotspots and trends of this field. The publications on the related topic might reveal variations in research activity and productivity, which could be classified into 3 phases. In the early stages, the integration of VR technology into stroke rehabilitation was minimal. The primary reasons for this included the high costs associated with VR systems and the nascent state of the technology [10,32]. VR hardware and software were not yet sophisticated or affordable enough to be widely adopted in medical settings [33]. During this period, research output remained relatively static, with few papers being published on the subject. With the reduced costs and the availability of high-quality technologies in the 2010s, there was a steady increase in the number of publications, reflecting the growing interest and investment in this area. The COVID-19 pandemic acted as a catalyst for the adoption of VR in stroke rehabilitation. With the need for remote health care solutions, telerehabilitation gained prominence [34,35]. VR, being a versatile and effective tool for remote rehabilitation, saw a dramatic increase in use and research interest. By 2021, the number of annual publications exceeded 100, indicating a significant surge in research activity. It is foreseeable that this research area will maintain its popularity in the near future.

In terms of the national distribution of researchers, more than one-third of the world’s countries have published papers on the application of VR technology for stroke rehabilitation. Notably, the United States, Italy, Canada, the United Kingdom, and Spain have emerged as predominant leaders in this field, significantly contributing to telemedicine research facilitated by VR. This dominance can be attributed to the substantial gross national product of these Western European and North American countries, which provides robust support for clinical research, in addition to the higher incidence and prevalence of stroke within these regions [36]. Furthermore, in the last five years, only two developing nations, China and Brazil, have made notable contributions in this domain. Particularly, Chinese ongoing investment in the application of novel smart technologies to health care management, along with the increasing availability of more portable, personalized, and affordable VR devices, is becoming increasingly viable with continuous innovations in smart technologies such as AI and the advent of the 5G era [37]. Nevertheless, the relatively low average citations per article for Chinese research indicates a need for improvement in the quality of their publications. The Chinese government is also actively seeking to enhance the quality of its research output in the future.

The analysis of researchers and institutions, as presented in Table 1 and Figure 4, reveals that the most active institutions are primarily from prominent universities in developed countries, particularly Canada and the United States. These institutions have substantial academic resources, evident in the authors’ co-occurrence network. As shown in Table 3, half of the top 10 researchers is from Italian institutions, yet their citation counts are lower than those of North American researchers due to the recency of their publications. The distribution of core journals publishing virtual reality therapy research for stroke highlights that the top 10 journals publish less than a third of the total papers, indicating a broad dissemination across many journals. The IF of these journals is generally modest, with only three having an IF of 5 or higher. This suggests that the research quality in this field needs improvement, necessitating increased international collaboration for higher-quality clinical research outcomes.

### Hotspots Analysis

Author clusters are instrumental in identifying significant research trends, offering insight into the past, present, and future hotspots within a specific scientific domain [38]. As depicted in Figure 5 , the authors responsible for the publication of 1171 papers can be categorized into 3 distinct groups. The leading contributors currently driving the field forward are Levin Mindy, Calabrò Rocco Salvatore, and Qiu Qinyin, respectively. This categorization not only highlights the dominant figures but also provides insight into the evolving dynamics and key influencers in the research area.

The team of Levin Mindy has been at the forefront of investigating the efficacy of VR in improving upper limb function in stroke survivors [39,40]. Their research found that both healthy volunteers and stroke survivors used comparable movement strategies when grasping and placing a ball within VR environments [41]. These findings suggest that VR can simulate realistic tasks and provide an immersive rehabilitation experience, making it a promising and effective tool for stroke recovery [41]. By replicating the mechanics of daily life tasks, VR environments offer a unique opportunity to bridge the gap between clinical therapy and real-world applications, potentially accelerating motor recovery and improving functional performance in stroke survivors [42]. The immersive nature of VR not only engages patients in a meaningful way but also helps overcome some of the limitations of traditional rehabilitation methods, particularly by offering repetitive and task-specific exercises that are critical for motor learning and neuroplasticity [43,44].

The research group led by Calabrò Rocco Salvatore investigates the neurophysiology effects of VR on individuals with stroke. Their studies revealed significant increases in alpha-band power in the occipital regions and beta-band power in the frontal regions of cognitively impaired individuals with stroke following VR-based cognitive rehabilitation [45]. These results highlight the potential of VR not only for enhancing motor function but also for influencing brain activity patterns associated with cognition and sensory-motor integration. Further expanding on this, their research demonstrated that robotic-assisted gait training in combination with VR can enhance lower limb function in stroke survivors with lower limb paresis. Notably, this approach activates key brain regions that are essential for gait control and rehabilitation, suggesting that VR may facilitate neuroplastic changes by stimulating the brain’s motor networks [46]. This dual benefit—improving both motor and cognitive recovery—positions VR as a powerful tool in stroke rehabilitation, capable of promoting neuroplasticity and accelerating recovery across multiple domains of function [45-47].

Qiu Qinyin’s research team has made significant strides in developing home-based VR rehabilitation systems designed specifically for patients with chronic stroke. Their empirical studies have demonstrated that patients can use this system with minimal supervision, achieving significant enhancements in upper limb functionality. By empowering patients to perform exercises at home, these home-based VR systems not only reduce the strain on health care systems but also provide patients with more flexibility, potentially leading to better adherence and outcomes in the long term [48]. Furthermore, the team has integrated haptically rendered VR with robotic assistance to facilitate upper limb functional training in stroke survivors [49,50]. Comparative analyses indicate that this combined modality yields superior clinical outcomes relative to conventional physiotherapy methods. The findings suggest that haptically rendered VR and robotics not only augment patient engagement but also enable more precise and effective rehabilitation exercises, thereby advancing the standard of care in stroke rehabilitation [49-51].

### Keywords and Trend Analysis

The cluster analysis of co-occurrence and burst keywords can identify frontier topics and emerging trends in a field. In the case of VR stroke treatment, the analysis highlighted that upper limb function is a primary research area and direction. According to Figure 7C, the evolution of topics over time can be divided into three phases:

1. Phase I (2001‐2006): Early literature focused on the therapeutic modalities and rationale for VR therapy in stroke treatment. Key topics included the use of VR environments, motor learning, and induced movement therapy [52,53]. Researchers also used functional magnetic resonance imaging to explore the association between VR therapy and brain injury [54].
2. Phase II (2007‐2014): This period saw a significant increase in the global use of VR for stroke rehabilitation, particularly for upper limb motor function and balance transfer [55]. The research expanded beyond initial therapeutic modalities to explore broader applications of VR in stroke recovery.
3. Phase III (2015‐2020): With advancements in VR technology, especially immersive VR, research designs became more standardized, incorporating randomized controlled trials. This phase marked an increased focus on the efficacy of VR in combination with traditional physiotherapy and occupational therapy [56,57]. Studies validating the effectiveness of VR therapies for neurological disorders, primarily through RCTs, emerged as a major trend.

Overall, the transition from exploratory studies to more rigorous and standardized research reflects the growing maturity and credibility of VR in neurorehabilitation. This progression underscores the importance of ongoing innovation in VR technology and the need for high-quality research to validate its clinical benefits.

### Strength and Limitations

This review is the first to comprehensively summarize current publications and development trends in VR for stroke from a bibliometric perspective. To ensure a systematic and thorough evaluation, the study collected 1171 related papers published over the last two decades from the WOSCC database. Using popular bibliometric tools such as Citespace and VOSviewer, the study conducted a quantitative analysis of VR therapy in the stroke field, focusing on data related to countries, institutions, authors, journals, citations, and keywords.

Despite the comprehensive approach, certain limitations of this work need to be acknowledged: First, due to limitations of the analysis tools, we only analyzed publications in the WOSCC. Although WOSCC offers robust coverage of high-impact journals, our exclusive reliance on this platform excluded potentially relevant studies from PubMed, Scopus, and Google Scholar, which may skew the geographical and methodological trends. Second, the language restriction to English-language publications in our inclusion criteria risks introducing publication bias, as significant research outputs in other languages were systematically excluded. Third, inherent limitations exist in our search strategy: only peer-reviewed articles and review papers were considered, thereby excluding potentially valuable academic books, conference proceedings, and gray literature. These methodological constraints underscore the necessity for future bibliometric investigations in VR for stroke rehabilitation to implement multi-database search architectures, incorporate multilingual literature, and develop more inclusive document type classification frameworks to provide a more inclusive and accurate representation of the research landscape in VR for stroke rehabilitation to provide a more inclusive and accurate representation of the research landscape in VR for stroke rehabilitation.

### Conclusion

This bibliometric study offers a comprehensive analysis of research trends in VR therapy for stroke rehabilitation over the past three decades. The data reveal a consistent upward trajectory in the number of studies, with North America and Western Europe leading in both publication volume and citation impact. The majority of these studies have been published in journals with relatively low IF, indicating a need for heightened focus on this area in future research. The author co-occurrence analysis identified 3 predominant clusters centering on neurophysiological effects of VR for stroke by Calabro RS, upper limb motor rehabilitation by Levin Mindy, and telerehabilitation of VR for stroke by Qiu Qinyin, respectively. The analysis also highlights the most recent research trends, which include the optimization of VR devices and the integration of VR with traditional physiotherapy and occupational therapy. These insights can guide future researchers in understanding the current focal points and potential directions for further development in VR therapy for stroke rehabilitation.

## Abbreviations

IEEE: Institute of Electrical and Electronics Engineers
IF: Impact Factor
IRCCS: Istituto di Ricovero e Cura a Carattere Scientifico
SCIE: Science Citation Index Expanded
VR: virtual reality
WOS: Web of Science
WOSCC: Web of Science Core Collection
